# A Novel Self-Activated Mechanism for Stable Liquid Transportation Capable of Continuous-Flow and Real-time Microfluidic PCRs

**DOI:** 10.3390/mi10060350

**Published:** 2019-05-28

**Authors:** Di Wu, Bing Shi, Bin Li, Wenming Wu

**Affiliations:** Changchun Institute of Optics, Fine Mechanics and Physics (CIOMP), Chinese Academy of Sciences, Beijing 100864, China; wudi16@mails.ucas.ac.cn (D.W.); shibing1105@foxmail.com (B.S.); libin17@mails.ucas.ac.cn (B.L.)

**Keywords:** self-activated fluidic mechanism, microdroplet, 3D microchannel, real-time PCR, house-made setup

## Abstract

The self-activated micropump capable of velocity-stable transport for both single-phased plug and double-phased droplet through long flow distance inside 3D microchannel is one dream of microfluidic scientists. While several types of passive micropumps have been developed based on different actuation mechanisms, until today, it is still one bottleneck to realize such a satisfied self-activated micropump for the stable delivery of both single and double-phased liquid inside long microchannel (e.g., several meters), due to the lack of innovative mechanism in previous methods. To solve this problem, in this article, we propose a new self-activated pumping mechanism. Herein, an end-opened gas-impermeable quartz capillary is utilized for passive transport. Mechanism of this micropump is systemically studied by both the mathematical modeling and the experimental verifications. Based on the flow assays, it totally confirmed a different pumping principle in this paper, as compared with our previous works. The $R^{2}$ value of the overall flow rates inside the 3D microchannel is confirmed as high as 0.999, which is much more homogeneous than other passive pumping formats. Finally, this novel micropump is applied to continuous-flow real-time PCRs (both plug-type and microdroplet-type), with the amplification efficiency reaching 91.5% of the commercial PCR cycler instrument.

## 1. Introduction

Microfluidics is a technique for precise control and manipulation of micro-scale fluids especially under submicron structures [1,2]. As the “heart” of the microfluidic system, micropump plays a significant role in fluidic transport. Due to the important functions of micropumps, many different principles have been designed to automate the transportation.

Depending on the power source, all micropumps can be divided into two categories: Externally-powered and self-powered micropumps. Additionally, all these micropumps have huge application prospects in a wide range of fields, such as drug delivery, blood transport, Chemical and biological analysis, electronic cooling, and so on [3,4,5].

Externally-powered micropumps (e.g., piezoelectric micropumps [6,7], syringe pump [8,9,10], thermopneumatic micropump [11], magnetic micropump [12], and acoustic micropump [13,14]) use off-chip power such as electricity, mechanics, magnetism, and acoustic to drive the flow of microfluidic. Benefiting from the stability of the external components and control system, these micropumps can precisely control the speed of the microfluidic. While these micropumps have prominent advantages, they are always giant in instrument size, and thus, it is hard to integrate the external-power micropumps into microfluidic devices to realize a miniaturized all-in-one set-up.

As a result, several types of self-powered micropumps have also been developed to realize the miniaturization and portability of the total microfluidic set-up. These self-powered micropumps generally use surface tension [15,16], evaporation pressure [17], hydrostatic force [18,19], gas diffusion/permeation [20,21], chemical/enzymatic reaction [20], or biophysics to induce the fluid flow in microfluidic devices.

In the last few years, to maintain and stabilize the flow velocity inside long microchannel (especially 3D configuration) during long term, we have proposed a self-powered micropump that depends on the permeability coefficient of the silicone or PDMS elastomer to control the flow rate [20,22,23,24]. While this type of passive micropump displays advantageous performance for stable sample transport, even inside four meters’ long microchannel concerning 3D channel-configuration and high temperature microenvironment, there is a non-negligible shortcoming associated with this method, which has not been solved. The self-powered flow is virtually automated by the gas permeability of the silicone [20,23] or PDMS wall [16,24], but the specified permeability coefficient of silicone or PDMS is determined by the size distribution of mini-pores in the elastomer, which usually varies from the fabrication process, curing conditions, and the production batch, and thus increases the difficulty in accurately controlling the passive flow rate.

In this paper, we have proposed a new method to achieve the self-powered micropump, which avoids the disadvantages of the micropump automated by the gas permeability of the silicone [20,24] or PDMS wall [16,24]. An end-opened quartz capillary is utilized to replace the end-blocked silicone tube for flow adjustment, which can dramatically increase the volume of liquid transported and the flow-duration. By changing the length and the inner diameter of the quartz capillary that connects the outlet of the microchannel, the speed of the microfluidic can be precisely controlled, and thus qualified for large volume of aqueous/oil phase transport through long microchannel, which was impossible in previous reports. By using the gas permeability of the quartz capillary to control the flow velocity, no liquid will flow into the quartz tube. Benefitting from this improvement, the flow velocity is more uniform and stable. The velocity is systemically studied by adjusting the length and inner diameter of the tail quartz capillary, and the inner pressure of the fluidic conduit as well. Finally, a house-made setup microreactor of continuous-flow real-time PCRs with the sample transported inside 3D spiral chip is realized, and it only relies on a single thermostatic heater for thermal cycle.

## 2. Principle

**Figure 1:**
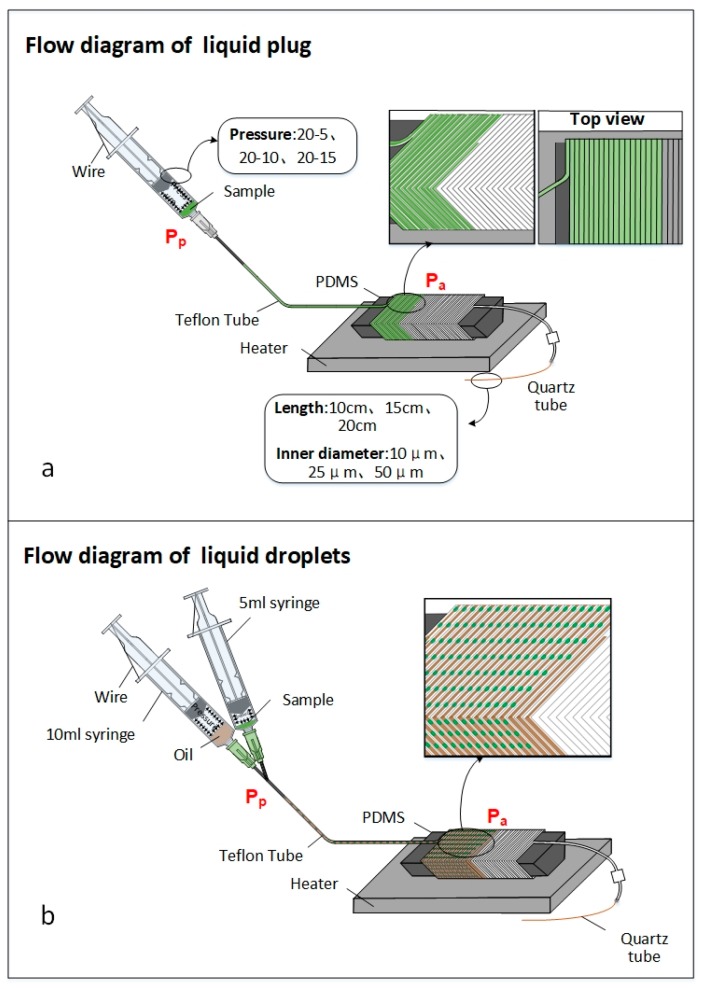
The principle of self-activated micropump applied to the end-opened system, where $P_{p}$ and $P_{a}$ represent the air pressures in the posterior and anterior ends of the reagent. (**a**) The self-activated plug flow and (**b**) the self-activated droplet flow.

Figure 1 shows a schematic illustration of the actuation mechanisms of the self-activated micropump applied to both the single-phased and the double-phased transport in the end-opened system. As we can see, it is based on the air permeability from the fluidic conduit to atmosphere through the tail quartz capillary. Thus, the permeation actually relies on the air passing through the hollow channel of the gas-impermeable quartz capillary.

Since the pressure of the compressed air captured inside the fluidic conduit is much higher than the atmospheric pressure, air molecules inside the microchip tend to penetrate to the ambient atmosphere through the hollow channel of quartz capillary. The air permeation only occurs at the outlet of the quartz capillary, which causes a decrease of the air molecules’ mole-number in the anterior end of sample plug. Based on Fick’s Law, this can be calculated by the following equation.

(1) $$G_{a}=\frac{DA_{ave}}{Z}\left(C_{Aia}-C_{Ao}\right)$$

(2) $$C_{Aia}=\frac{P_{a}}{RT}$$

(3) $$C_{Ao}=\frac{P_{Ao}}{RT}$$

(4) $$A_{ave}=a\pi r_{q}^{2}$$

where $G_{a}$ is diffusion rate, D is equivalent diffusion coefficient, $C_{Aia}$ is inner air molecule concentration in the anterior end of sample plug, $P_{a}$ represents the air pressures in the anterior end of the reagent, $C_{Ao}$ is air molecule concentration of ambient atmosphere, Z is diffusion distance and can be represented by the length of quartz capillary, $A_{ave}$ is the average diffusion area,$\;r_{q}$ is the diameter of the quartz capillary, a is the correction coefficient between an actual condition and an ideal condition.

The equivalent diffusion coefficient of quartz capillary is only determined by the dimension of its hollow channel, but not variable to the distribution of pore-size in the wall of PDMS or silicone elastomer as previously reported.

As shown in Figure 1a, $P_{p}$ represents the air pressures in the posterior end of the reagent, while $P_{g}$ represents the pressure gradient imposed in the reagent, and can be calculated by the following equation:

(5) $$P_{a}=P_{p}-P_{g}$$

For an easier modeling of this new self-activated pumping system, an equivalent condition forms during the microfluidic transport, when the fluidic-flux and air-molecule-penetration is the same with each other, producing the constant pressure$\;P_{a}.$

If the microchannel is column-configuration, then

(6) $$v=\frac{Da\pi r_{q}^{2}(P_{p}-P_{g}-P_{o})}{RTZr^{2}}$$

where v is the velocity of the microfluidic, r is the radius of the microchannel. Easily seen, the flow rate decreases if the radius of the microchannel increases.

Since atmospheric pressure$\;P_{o}$ is constant, and$\;(P_{p}-P_{g})$ is also kept constant, hence the flow rate is v is constant, which can also be represented by the following equation,

(7) $$v=\frac{Dar_{q}^{2}}{RTr^{2}}\frac{dP}{dZ}$$

where dP/dz is the pressure gradient across the hollow channel of the quartz capillary connecting the microchip to the atmosphere.

Based on the above equation, we can predict that the flow velocity increases as the quartz capillary’s length (Z), inner diameter ($r_{q}$), and inner compressed pressure ($P_{a}$, $P_{p}$) increase.

Both the flow of liquid plug and liquid droplet can be realized by this self-activated micropump. As we can see in Figure 1b, before we set the pressure by pressing the syringe, the air pressure all through the fluidic conduit is the same. After the reagent and the oil phase (HFE 7500) are added into the insert tips of the 5 mL and 10 mL syringes, the pressure of each syringes maintains the same during the flow process. If the volume ratio between the 10 mL and 5 mL syringes is n:1, then the velocity ratio between the oil and the reagent is also n:1. If the velocity of the oil and the reagent are $v_{o}$ and $v_{r}$, it can be calculated that $v_{o}=nv_{r}$. Therefore, the reagent is divided into droplets by the oil, with the micropump here keeping a steady and homogeneous transport velocity when delivering reagents through a very long microchannel.

Noticeably, herein the principal depends on ideal gas but not the actual gas, which can cause a difference between the principle prediction (ideal condition) and measured experimental results (actual condition). In addition, the diffusion mechanism mainly depends on the Fick’s Law and thus is only available for very thin capillary. If the diameter of the capillary is huge, e.g., bigger than 100 μm, then other regularity such as the convection mass transfer will become the dominant effect. Under such conditions, other factors such as Peclet number of air transport can be relatively high (i.e., *Pe* > 2), and resulted in relatively higher flow rate than the air diffusion rule here. Since a very high flow rate is non-applicable for the downstream continuous flow PCR filed here, the flow rule activated by the convection mass transfer will not be discussed in this paper.

## 3. Experimentation

### 3.1. Flow Experiment

During the flow analysis, the device set-up consisted of two 20 mL syringes, one quartz capillary, one clamp, one 27G needle, one 5 cm-long silicone tube (OD = 3 mm, ID = 1 mm), one iron wire and Teflon tube with its inner and outer diameters to be 0.3 mm and 0.6 mm, respectively. Next, 30 µL to 50 µL green ink was added to the syringe, and then the Teflon tube was blunt-ended by a clamp. This system was activated by pressing the syringe from the scale 20 mL to a certain scale, and was fixed by the iron wire. At the end of the system, between the Teflon tube and the quartz capillary, we added a 5 cm-long silicone tube to contain the liquid in case it might flow into the quartz capillary and affected the stability of flow velocity.

After the devices have been installed, the green ink was moved to the pin of the syringe. After removing the clamp, the ink flew spontaneously into the Teflon tube under the consistent pressure inside the system.

We designed three groups of experiments to explore the flow of liquid plug under three different conditions.

1. By changing the inner diameter of the tail quartz capillary

This experiment was carried out with three different quartz capillaries which had different inner diameters of 10 µm, 25 µm and 50 µm, respectively. To maintain the other parameters the same, the length of the quartz capillary was set to be 10 cm, and the 20 mL syringe was pressed from the scale of 20 mL to the scale of 10 mL. Each experiment was operated for three times.

2. By changing the pressure of the syringe

This experiment was carried out by varying the inner pressures of the syringe. The pistons of 20 mL syringe was pushed from scale 20 mL to scale 5 mL, 10 mL, and 15 mL, respectively. The quartz capillary’s inner diameter was 25 µm, and its length was 15 cm. Each experiment was operated for three times.

3. By changing the length of the tail quartz tube

This experiment was carried out with three different lengths of quartz capillaries, namely 10 cm, 15 cm and 20 cm. And the quartz capillary’s inner diameter was 25 µm. The 20 mL syringe is pressed from the scale of 20 mL to 10 mL. Three groups of experiments were operated, with each experiment repeated for three times.

### 3.2. Application in Continuous-flow On-chip PCRs

After the flow experiments, we tested the self-activated micropump system for applications of continuous-flow on-chip PCRs. Altogether, we prepared two sets of experiments to evaluate our system, the reaction of plug-based PCR and the droplets-based PCR.

1. The plug-based PCR

**Figure 2:**
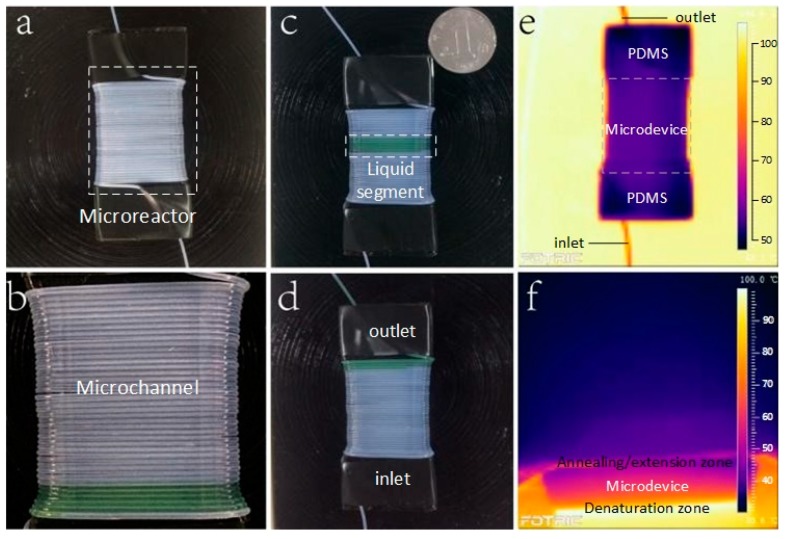
The set-up for plug-based continuous-flow on-chip PCR (**a**) with the green-color sample flowing at the start (**b**), the middle (**c**), and the end (**d**) of the fluidic conduit. (**e**) The top view and (**f**) the side view of the thermal infrared image of the trapezoidal PDMS block.

The Teflon tube was twined around a trapezoidal PDMS block for 40 rings instead of a syringe. The widths of the top and the bottom surface of the trapezoidal PDMS block are 20 mm and 10 mm, with the height and length set to be 8.5 mm and 50 mm, respectively. We cover the block with black tape for the purpose of measuring its surface temperature by IR camera. The 20 mL syringe is pressed from the scale of 20 mL to the scale of 10 mL. The quartz capillary’s inner diameter is 25 µm, and its length is 15 cm. All the connections were sealed with silicone adhesive. As shown in Figure 2c, we put a coin at the upper right corner of the device to visually show its total size.

A single heater can achieve the thermal cycle requirement by controlling the temperature gradient of the PDMS block. In order to make sure the temperatures of the upper and lower surfaces were suitable for continuous flow microfluidic PCRs, we used an infrared (IR) camera (Fotric 220, ZXF Laboratory, Allen, TX, USA) to monitor the temperature. The tube wrapped PDMS block was placed on the top of the 95 °C heater. Through a series of parallel experiments, we adopted to make the height of the PDMS block to be 8.5 mm, so that the temperature of the upper surface reached 60 °C, which is suitable for the PCR reagent.

To prove that the micro-device can be used to continuous flow PCRs, we used a commercial PCR cycler (CFX Connect, Bio Rad, Hercules, CA, USA) as reference. By comparing the product of the two devices, we could verify the function of our system.

The PCR Reagents contained a buffer composed of 1X SYBR Premix Ex Taq II, 0.075U µL^−1^ TaKaRa EX Taq, 0.6 mg·mL^−1^ BSA (AS25483, AMEKO, Dalian, China), 1 µM forward and reverse primers, and 0.3326 ng/µL template. The primer sequences were as follows:

5′ ACA GAA TCA GGG GAT AAC GCA GGA AAG AAC A 3′ (forward);
5′ GTC AGG GGG GCG GAG CCT ATG GAA AAA C 3′ (reverse).

The gene of pGEM-3Zf (+) fragment was inserted into pUC57-Kan plasmid vector (Genewiz, Suzhou, China) by recombinase, and it was further used as the PCR target. After the PCR reaction, agarose powder (V900510; Sigma-Aldrich, Shanghai, China; www.sigmaaldrich.com, MO; 2%), DL2000 DNA marker (50 × 250 µL, Peking Jialan Biotechnology Co., Ltd., Beijing, China), 0.5 × TBE buffer (PH1755, Phygene, Fuzhou, China), and Nucleic Acid GelStain (KeyGEN BioTECH, Nanjing, China) were applied to analyze the amplification result.

2. The droplet-based PCR

**Figure 3:**
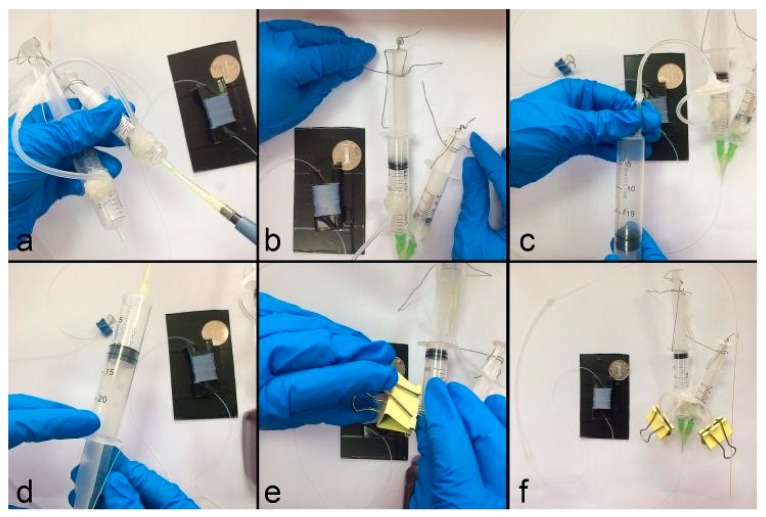
The assembly process for the set-up of the droplet-based continuous-flow on-chip PCR. (**a**) The injection of the liquid and oil phases; (**b**,**c**) the joint of the device; (**d**,**e**) pressure setting; (**f**) complete assembly of droplet formation system.

We slightly modify the previous plug-based PCR system as shown in Figure 3. Instead of using one syringe to produce pressure, the droplets generation unit was activated by three pressurized syringes with original volumes of 5 mL, 10 mL, and 20 mL, respectively.

Before assembling the device, two holes were drilled (Jeben, Yongkang, China) at the 1 mL graduation of the 5 mL syringe and 4.5 mL graduation of 10 mL syringe. Three silicone tubes (ID = 1 mm, OD = 3 mm, length = 50 mm) were used to interconnect the 5 mL, 10 mL and 20 mL syringes by one T-connect (ID = 1 mm, OD = 1.6 mm). The oil-phase and the aqueous-phase were added to 10 mL and 5 mL syringes, which were then connected to the inlet of Teflon tube by two 34G needles, with the junction sealed by hot melt adhesive. The pistons of the 5 mL syringe and the 10 mL syringe were fixed at the scale of 2 mL and 9 mL by iron wires. Next the 20 mL syringe was pushed from the scale of 20 mL to 10 mL, to produce an inner pressure and fixed by a third iron wire. Then, we used two clamps to blunt the two silicone tubes between the T-connect and 5 mL, 10 mL syringes. The two syringes can be placed either vertically or obliquely to make both the oil and the reagent flow downward spontaneously [25].

**Figure 4:**
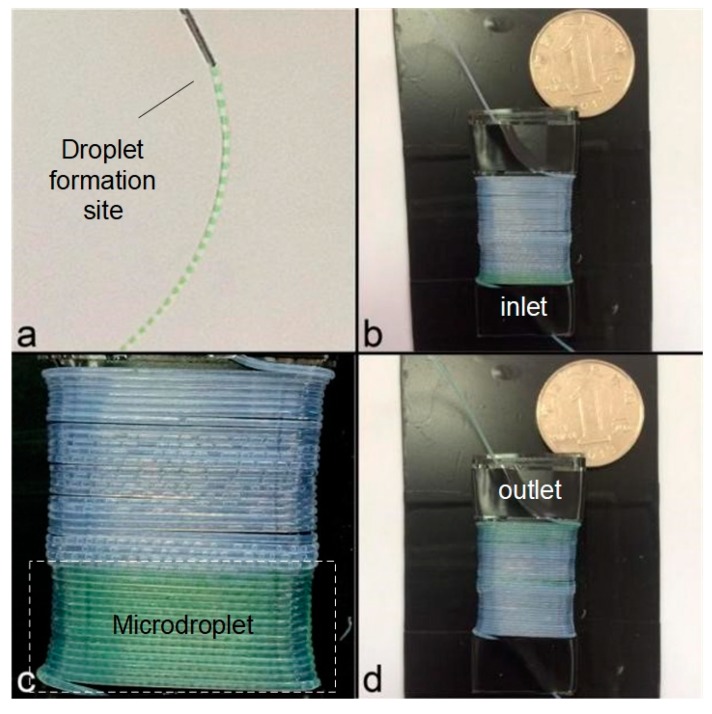
The droplet-based continuous-flow on-chip PCR with the green-color sample flowing at the inlet (**a**), the start (**b**), the middle (**c**), and the end (**d**) of the fluidic conduit.

As shown in Figure 4a, the droplets were automatically generated and transported inside the Teflon tube. The same PCR chip as previous plug experiment was used, with the length and inner diameter of the tail quartz capillary set to be 20 cm and 25 µm, respectively.

We used a single heater to achieve the temperature requirement of the PDMS block wrapped with Teflon tube. The Fluorinated oil (HFE 7500) was used as the oil phase. The other reagents were the same as the plug-based PCR experiment.

### 3.3. Real-time Fluorescence Detection

To prove the microdevice can be used to continuous-flow real-time PCRs, a commercial real-time qPCR (CFX Connect, Bio Rad) was used as reference. By comparing the fluorescence intensity and the products gained from the two devices, the performance of the micro-device could be verified.

PCR Reagents contained a buffer composed of 1X SRBR Premix Ex Taq II, 0.075 U μL^−1^ TaKaRa EX Taq, 0.3 mg·mL^−1^ BSA (AS25483, AMEKO), 1 μM forward and reverse primers, and 10^8^ to 10^5^ copies μL^−1^ DNA template. The primer sequences were as follows:

5′ TAC AGA CAA TCC CCG ACC GA 3′ (forward)
5′ GCC AAG TGT TAG CCC CAT CC 3′ (reverse).

The gene of H7N9 was inserted into pUC57-Kan plasmid vector (Genewiz, Suzhou, China) by recombinase, which was further used as the PCR target.

The fluorescence detection unit consisted of 48 Watts LEDs array (XPE60W, Cree, NC, USA), a digital camera (Canon EOS 7D, Tokyo, Japan), a 480 nm and a 520 nm narrowband filter (Xintian bori, Beijing, China). The 480 nm narrowband filter was fixed in front of the LEDs array to offer the excitation light, and the 520 nm narrowband filter was fixed in front of the camera lens. The LEDs array was controlled by cycle relay and lighted for 5 s every 50 s, and the camera connected to the laptop automatically took photos when the LEDs array was on.

The fluorescence images were captured by aforementioned digital camera, with the parameters set as follows: F = 2.8, M = 1/20, and ISO = 2000. The images obtained was processed by the software Image J (version 1.48, National Institutes of Health, Berlin, Germany), to distinguish the light from surroundings conditions. The grayscale value was regarded as the background noise. By counting the light intensity of the liquid plug in each cycle, the fluorescence intensity curve of PCR amplification could be obtained.

## 4. Results and Discussion

### 4.1. Flow Analyses

**Figure 5:**
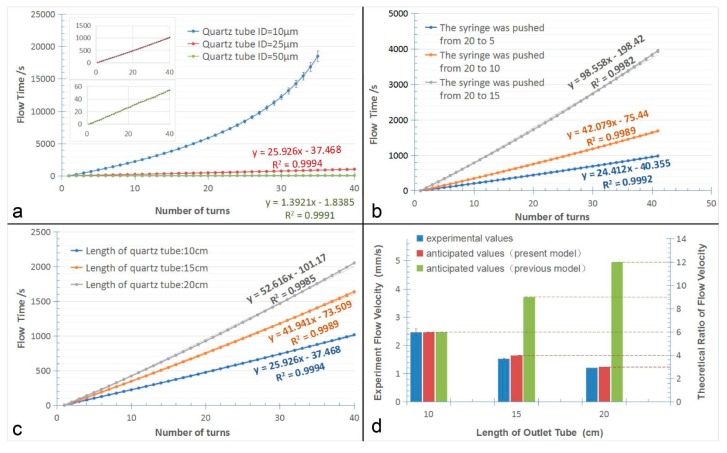
The self-activated flow influenced by (**a**) inner diameter of the tail quartz tube, (**b**) the pressure, and (**c**) the length of the tail quartz tube. (**d**) The relationship between the flow rates and the length of the tail quartz tube anticipated from two mechanisms, and the assays of measured flow velocities. (The previous model is based on our previous works [20,22,23,24], and the present model is our method, illustrated in this article.).

As shown in Figure 5a, the total running time were 18,483 s, 1016 s, and 62 s, when the inner diameters of the quartz capillary were respectively 10 µm, 25 µm, and 50 µm. When the quartz capillary’s inner diameter was 10 µm, the flow resistance was so significant that severely affected the flow rate, causing the velocity of the liquid was extremely slow, and thus, the retention time increased rapidly after 20 rings. On the contrary, the flow velocity of the system was too fast when the inner diameter of tail quartz capillary was 50 µm, which was around one or two seconds per circle. But when the inner diameter of the quartz capillary was 25 µm, the flow velocity and the flow time per circle were suitable for the PCR reagents to react, and the linearity of the curve was also better than the other two, with the $R^{2}$ value calculate to be as high as 0.9994. The results suggested that the flow rate of liquid could be precisely controlled by changing the inner diameter of the quartz capillary connected to the end of Teflon tube. For tail quartz capillary with the inner diameter of 50 µm, we estimated both the diffusion mechanism and the convection mass transfer affect the flow, and thus, the flow rate is almost 16 times of the tail quartz capillary with the inner diameter of 25 µm, much bigger than the prediction only determined by the diffusion mechanism, as discussed in the principle part.

Next, the results in Figure 5b showed the effect of the syringe pressure on flow rate. The inner diameter and length of the quartz capillary used in this experiment were 25 µm, and 15 cm, respectively. Obviously, the flow velocity of liquids was stable and increased as the pressure increased, and the total running time were 978 s, 1686 s and 3945 s when the syringe was respectively pushed from the scale of 20 mL to 5 mL, 10 mL and 15 mL. The results suggested that the flow rate of liquids could be adjusted by changing the pressure of the syringe. Based on the principle, the flow rate increased as the inner pressure of the fluidic conduit increased. When the syringe was respectively pushed from the scale of 20 mL to 5 mL, 10 mL, and 15 mL, the inner pressure can be calculated to be 4 atm, 2 atm and 1.33 atm if the air was considered to be ideal gas. Correspondingly, the ratio of flow rate between three systems was measured to be 5.37:2.29:1.33, implicating a same change-tread with theoretic prediction. Due to the difference between the ideal gas and the real gas, the difference between principle predication and the experiment assays are inevitable.

Finally, the results in Figure 5c were obtained by analyzing quartz capillaries with the same inner diameter of 25 µm, but with different lengths of 10 cm, 15 cm and 20 cm, respectively. Obviously, the flow rate of liquid was relatively stable and decreased as the length of the Teflon tube increased. The average running time of the liquid flow were 26 s, 42 s, and 53 s, respectively. The results showed that the length has great influence on liquid velocity. As demonstrated in Equation (6) of the principle, longer quartz capillary caused bigger diffusion length (Z), and thus decreased flow rate. Based on Equation (6), the velocity ratio of the three systems should be 6:4:3 if the lengths of quartz capillary were set to be 10 cm, 15 cm and 20 cm, respectively. And as we can see in Figure 5d the experimental results verified velocity ratio between the three systems should be 6:3.72:2.94, which was close to the novel principal in this paper. It also proved here that the pumping principle was totally different from our previous works relying on the permeability coefficient of the end-blocked gas-permeable silicone or PDMS wall according to the experimental results in Figure 5d. In our previous work, the flow rate is proportional to the tube length that connected to the outlet of fluidic conduit, but herein it displays an inversely proportional relationship.

### 4.2. Results of Plug-based Continuous Flow PCRs and Droplet-based Continuous Flow PCRs

As a proof of concept, this novel micropump was firstly applied to plug-based continuous flow PCRs in the end-opened system. The Teflon tube wrapped PDMS block should be placed on the single heater for at least 30 min to reach the set temperature before sample injection. To make sure the surface temperatures corresponded to the annealing, extension and denature temperature of PCRs, the denature temperature was adjusted to approximately 95 ± 0.8 °C (CV = 0.8%, n = 10), and the annealing temperature was adjusted to approximately 65 ± 0.7 °C (CV = 0.6%, n = 10).

The reaction condition in the commercial qPCR cycler is set as mentioned above, with the annealing stage and the denature stage set to be 30 s and 10 s, respectively. Meanwhile, the flow velocity of the reagent in the Teflon tube wrapped PDMS block is approximately 42 s per cycle. Due to the shape of the PDMS block, the 95 °C denature temperature lasted for 9 s, and the 65 °C annealing temperature lasted for 18 s. Besides, the extension stage lasted for about 15 s, which corresponds to the PDMS block side walls.

**Figure 6:**
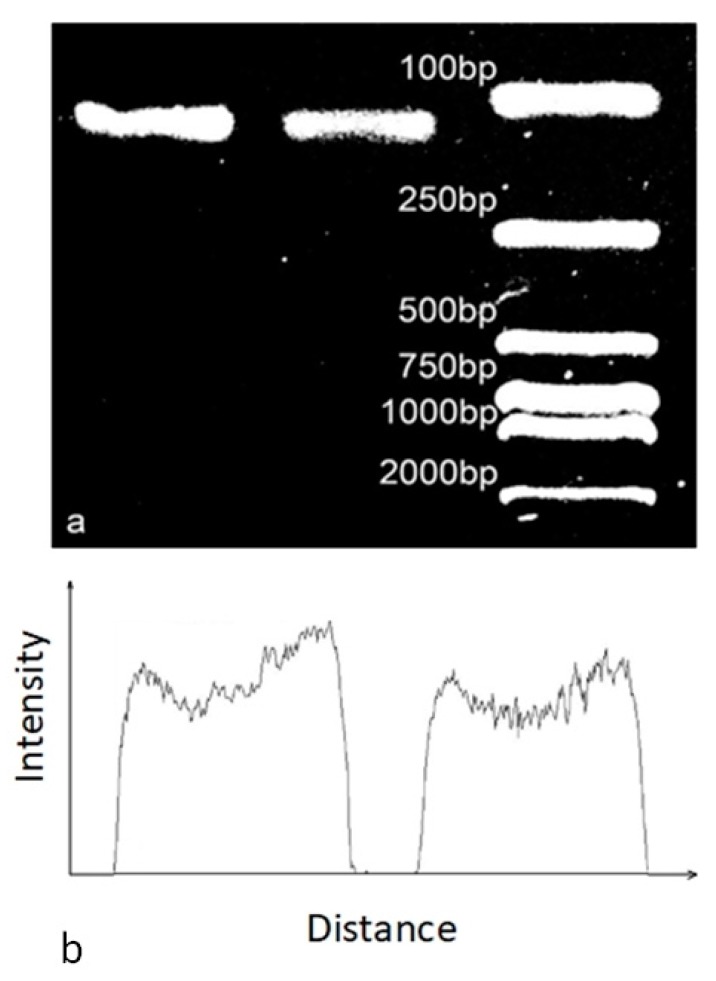
Electrophorogram and intensity diagram of contrast PCR experiments. (**a**) Electrophoretic results by commercial cycler (**left**), electrophoretic results by microdevice (**middle**) and the ladder (**right**). (**b**) The intensity diagram of commercial cycler PCR electrophoretic results (**left**) and microdevice PCR electrophoretic results (**right**).

After the reaction, the reagents gained from the microdevice and qPCR cycler were tested by agarose gel electrophoresis. The results were shown in Figure 6.

The result showed in Figure 6 demonstrated that the plug-based amplification efficiency of the microdevice was similar to the commercial qPCR cycler. By calculating the average gel intensity of targets, the amplification efficiency of the microchip reached 85.7% of commercial qPCR cycler, which suggested this novel self-powered micropump could be applied to plug-based continuous flow PCRs and the results were similar to commercial qPCR devices.

This novel self-powered micropump was further applied to droplet-based continuous flow PCRs. The reaction condition in the commercial qPCR cycler is set the same as the plug-based experiment. Meanwhile, the flow rate of the reagent in the Teflon tube wrapped PDMS block is approximately 40 s per cycle.

**Figure 7:**
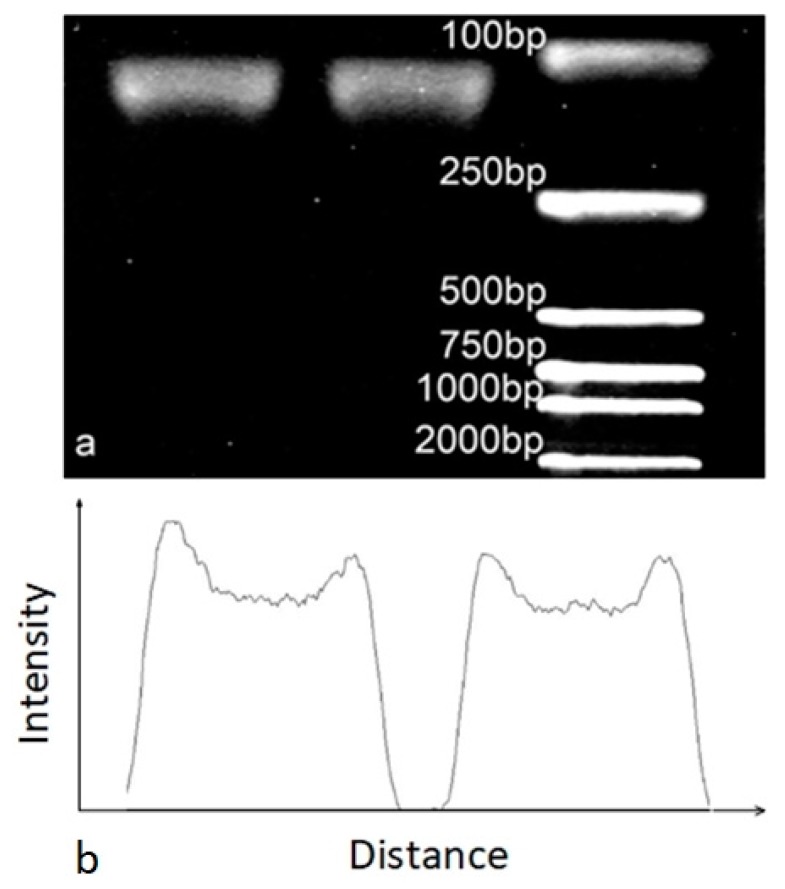
Electrophorogram and intensity diagram of the liquid droplets’ PCR experiment. (**a**) Electrophoretic results by commercial cycler (**left**), electrophoretic results by microdevice (**middle**) and the ladder (**right**). (**b**) The intensity diagram of commercial cycler PCR electrophoretic results (**left**) and microdevice PCR electrophoretic results (**right**).

Figure 7 demonstrated that droplets-based amplification efficiency of the microdevice was also similar to the commercial qPCR cycler. And the contrast amplification efficiency reached 91.5%. We assumed that Fluorinated oil can keep the droplet away from the inner wall of the Teflon tube, which increased the efficiency of the reaction.

Based on the aforementioned flow analyses, it proved the passive micropump can stably transport both the single-phased and the double-phased liquid inside several meters’ long microchannel of 3D configuration.

### 4.3. Results of the Real-time Fluorescence Detection

**Figure 8:**
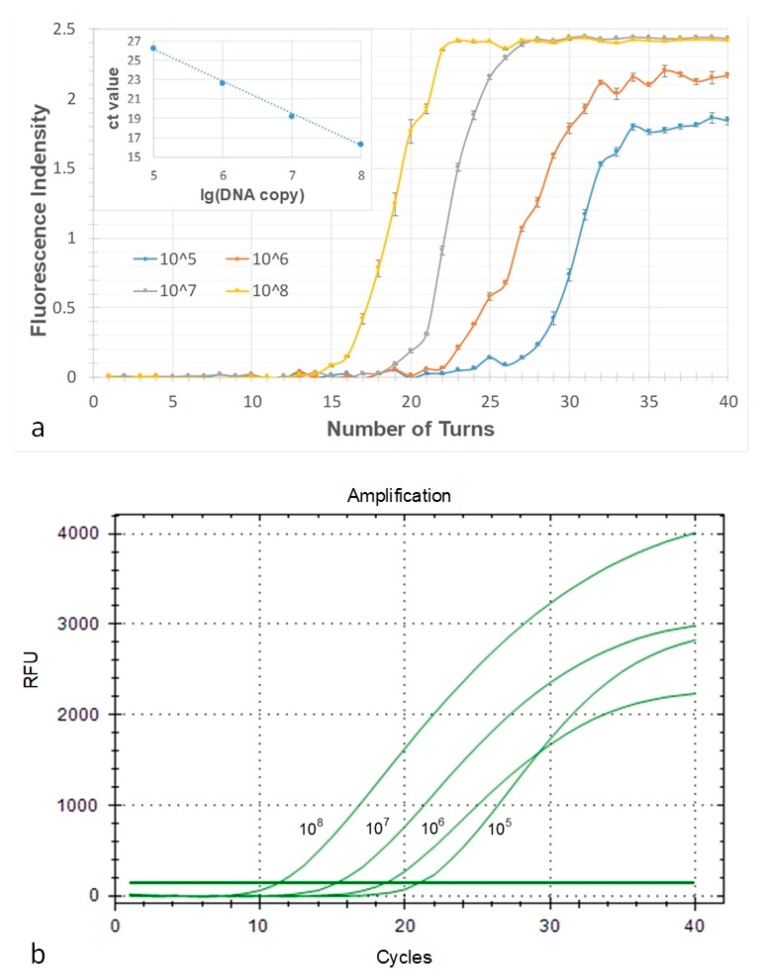
Results of real-time fluorescence detection. (**a**) The amplification curves gained from the fluorescence images of serial diluted DNA molecules of the microdevice. (**b**) Amplification curves from the commercial Real-time PCR amplification system.

To find out the PCR amplification efficiency of the novel system, four ordered serially diluted genes from 10^8^ to 10^5^ copies/μL were tested in both the micro-device and commercial qPCR cycler (Bio Rad). The images were obtained by digital camera (Canon EOS 7D). The results were shown in Figure 8.

As shown in Figure 8, dramatic decreased fluorescence signal could be seen as concentration of DNA template decreased from 10^8^ to 10^5^ copies μL^−1^. The fluorescence intensity curves shown in Figure 8a were obtained from camera images, and the software Image J was used to analyze fluorescence intensity of each image. The Ct values of the four curves were calculated to be 16.35, 19.22, 22.67 and 26.21. The results run by commercial qPCR were shown in Figure 8b, with the Ct values of 11.28, 15.28, 18.64 and 20.98, respectively. According to Figure 8, we could find that the trend of the four curves in the former image were similar with the latter, which suggested that the novel micropump could be applied to self-activated continuous-flow and real-time PCRs, and the results were similar to commercial qPCR devices.

## 5. Conclusions

We introduced a novel mechanism for self-powered liquid transport in the end-opened microchip. It provided a new approach to control the velocity of liquid conveniently by adopting the gas permeability of the quartz capillary. The flow assays confirmed a good coincidence between the theoretical formula and the experimental results. Furthermore, herein a totally contrary regularity with previous mechanisms [26,27,28,29] was also confirmed through the assays between the flow rate and the length of outlet-tube. In contrast with most other passive micropumps which have been developed until today, the passive micropump here displays the superior pumping performance concerning good bubbles’ suppression under extreme microenvironment of high temperature (95 °C), and thus, capable of complicated applications such as the house-made setup of plug/droplet-based continuous-flow real-time on-chip PCRs. In future research, we are going to upgrade this system to make it more controllable, and to apply it to wider fields.
